# Oronasal Fistula Closure and Defect Reconstruction: Two Case Reports Using Periodontal Plastic Surgery Principles

**DOI:** 10.1002/cre2.914

**Published:** 2024-07-07

**Authors:** Gerardo Chacon, Abdusalam Alrmali, Obada Mandil, Héctor Rodriguez, José Rodriguez, Anas Al‐misurati, Hom‐Lay Wang

**Affiliations:** ^1^ Private Practice Medellin Colombia; ^2^ Department of Periodontics and Oral Medicine, School of Dentistry University of Michigan Ann Arbor Michigan USA; ^3^ Department of Oral Pathology, Oral Medicine and Oral and Maxillofacial Surgery School of Dentistry University of Tripoli Tripoli Libya; ^4^ Private Practice Barquisimeto Venezuela; ^5^ Department of Periodontics, School of Dentistry University of Zawia Zawia Libya

**Keywords:** aesthetics, cleft lip and palate, dental, oral fistula/surgery, oronasal fistula, periodontics

## Abstract

**Objectives:**

Oronasal fistulas are common sequelae following cleft lip and palate surgery and can significantly impact a patient's quality of life. They result from various factors, including surgical techniques, tissue management, and patient‐specific factors. This case report explores the modern approach to oronasal fistula closure using periodontal plastic surgery principles.

**Materials and Methods:**

The report presents two cases of patients with oronasal fistulas due to previous maxillofacial surgical intervention. These patients underwent microsurgical procedures that involved partial flap thickness preparation of the fistula areas, the use of connective tissue grafts from the palate, and meticulous suturing techniques to ensure graft integrity. The procedures were performed in stages, and postoperative care was provided.

**Results:**

Both cases demonstrated successful fistula closure and graft survival. The patients reported improvements in breathing, speech, aesthetics, and quality of life. The second case also included guided bone regeneration and implant placement.

**Conclusions:**

Oronasal fistulas resulting from maxillofacial surgery can be effectively treated using periodontal plastic surgery techniques, significantly improving patients' quality of life and aesthetic outcomes. This approach represents a valuable addition to the existing repertoire of oronasal fistula closure methods.

## Summary

- In this case report, we present two adult patients who underwent oronasal fistula closure procedures applying principles of periodontal plastic surgery, highlighting successful outcomes and the potential application of these techniques in similar cases.

## 1 Introduction

Oronasal fistulas are a common complication following cleft lip and palate surgery, occurring in a range of 4%–35% of cases (Cohen et al. 1991; Honnebier et al. 2000; Li, Yin, and Song 2015; Wilhelmi et al. 2001). The likelihood of this complication increases when it is associated with factors such as excessive tension on the surgical flap, trauma, infection, perforation, or a history of palatoplasty (Cohen et al. 1991; Honnebier et al. 2000; Li, Yin, and Song 2015; Wilhelmi et al. 2001). Additionally, the occurrence of fistulas and oronasal communications is influenced by several surgical factors, including the surgeon's expertise, level of training, surgical techniques employed, postoperative care, administration of antibiotics, the patient's nutritional status, the age at which the surgery is performed, the specific type of defect, and the collaborative efforts of the medical team managing the case, among other variables (Aznar et al. 2015; Bonanthaya et al. 2016). Patients commonly report various issues, including the passage of food into the nasal cavity, changes in speech, bad breath, regurgitation, breathing difficulties, and a decline in their overall quality of life. These problems can have a particularly significant negative impact on the psychological and social well‐being of children and young individuals (Li, Yin, and Song, 2015; Pollard et al. 2021).

From an embryological perspective, the development and fusion of the palate, jaws, nose, and lips involve the formation of distinct regions. These processes are regulated by cell signaling events occurring between the fourth and 12th weeks of gestation. The outcome depends on the precise location, maturation, and eventual fusion of these structures. The neuroectoderm, originating from the neural crest, migrates to form the five facial structures in the frontal and visceral regions. Any disruptions in these processes can result in variations that are closely associated with the defect's location, direction, depth, and complexity. Consequently, various treatments have been categorized and tailored to address these differences (Smith et al. 2007).

These treatments encompass a range of approaches, including obturators, rotational flaps, lingual flaps, displaced flaps, palatal flaps, buccal myomucosal flaps, tissue grafts, local flap techniques, tissue expanders, cartilage grafts, skin grafts, three‐layered closure techniques, acellular dermal matrix, and alveolar ridge management, among others (Abdel‐Aziz 2010; Abuabara et al. 2006; Li, Yin, and Song 2015; MacLeod et al. 1987; Robertson et al. 2008; Simpson et al. 2019; Steele and Seagle 2006; Tunçbilek et al. 2012; Zhang et al. 2014). Nonetheless, incorrect flap management and rotation can lead to the formation of an epithelialized tract, potentially resulting in a new fistula, particularly when associated with premaxilla removal. To the best of the authors' knowledge, no technique has been described to address the closure of such fistulas using various types of grafts in the context of periodontal plastic surgery with an emphasis on aesthetics. The subsequent case report provides a detailed account of this innovative technique.

## 2 Materials and Methods

### 2.1 Clinical Presentation

The following case report presents a bilateral fistula in the buccal vestibule, because of previous maxillofacial surgery, for cleft lip and palate correction, which included amputation of the premaxilla. The original study protocol underwent rigorous ethical scrutiny and received approval from the Ethics Committee, specifically for data collection and the sharing of photos. Patients who participated in the original study provided explicit written consent. As an essential aspect of ensuring patient privacy and confidentiality, specific patient files and data were meticulously safeguarded. It is worth highlighting that the original analysis adhered to the ethical principles outlined in the World Medical Association Declaration of Helsinki, ensuring that the study was conducted with full regard for the rights and well‐being of the participants.

## 3 Case 1

### 3.1 Clinical Presentation

**Figure 1:**
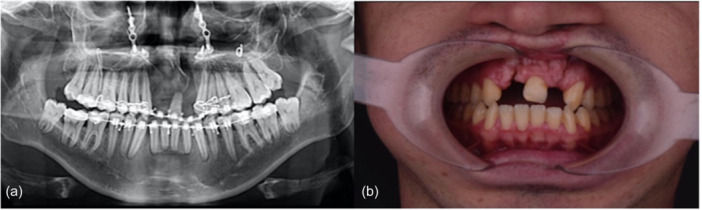
(a) Radiographical and (b) clinical baseline images showing previous surgical repair of bilateral cleft lip and palate with oronasal fistula.

A 32‐year‐old nonsmoker in good health was referred for a consultation to the Maxillofacial Surgery Service at the International Hospital in Barquisimeto, Venezuela. The patient's concerns were primarily associated with bilateral fistulas that had resulted from cleft lip and palate surgery performed during infancy (between the ages of 3 months and 3 years). These fistulas had given rise to a range of issues, including oronasal communication, the passage of food into the nasal cavity, unpleasant odors, regurgitation, speech difficulties, respiratory problems, and cosmetic concerns (Figure 1a,b). The patient agreed to proceed with the recommended microsurgical treatment plan, which would be conducted in stages.

### 3.2 Surgical Case Management

In the first stage, following thorough bacterial plaque control, microsurgical preparations were performed by creating partial‐thickness tunnels within each of the fistulas located in the buccal vestibule. These tunnels extended from the base and wings of the nose and included a tooth on both the mesial and distal sides of the defect. Microsurgical blades (No. 003‐004, MJK Instruments, France) were employed for this procedure. A connective tissue graft harvested from the palate was then placed inside these tunnels, with absorbable sutures (polyglactin (Vicryl 7‐0), Assut sutures, Switzerland) used to secure it from one side of the defect to the other. Finally, the outer edges of the surgical bed, which had been previously de‐epithelialized, were approximated and sutured using 6‐0 Polypropylene (Assut sutures). The patient was provided with postsurgical instructions, which included rinsing with saline solution three times a day, taking 500 mg of amoxicillin every 8 h, and consuming 600 mg of ibuprofen every 8 h for 1 week.

**Figure 2:**
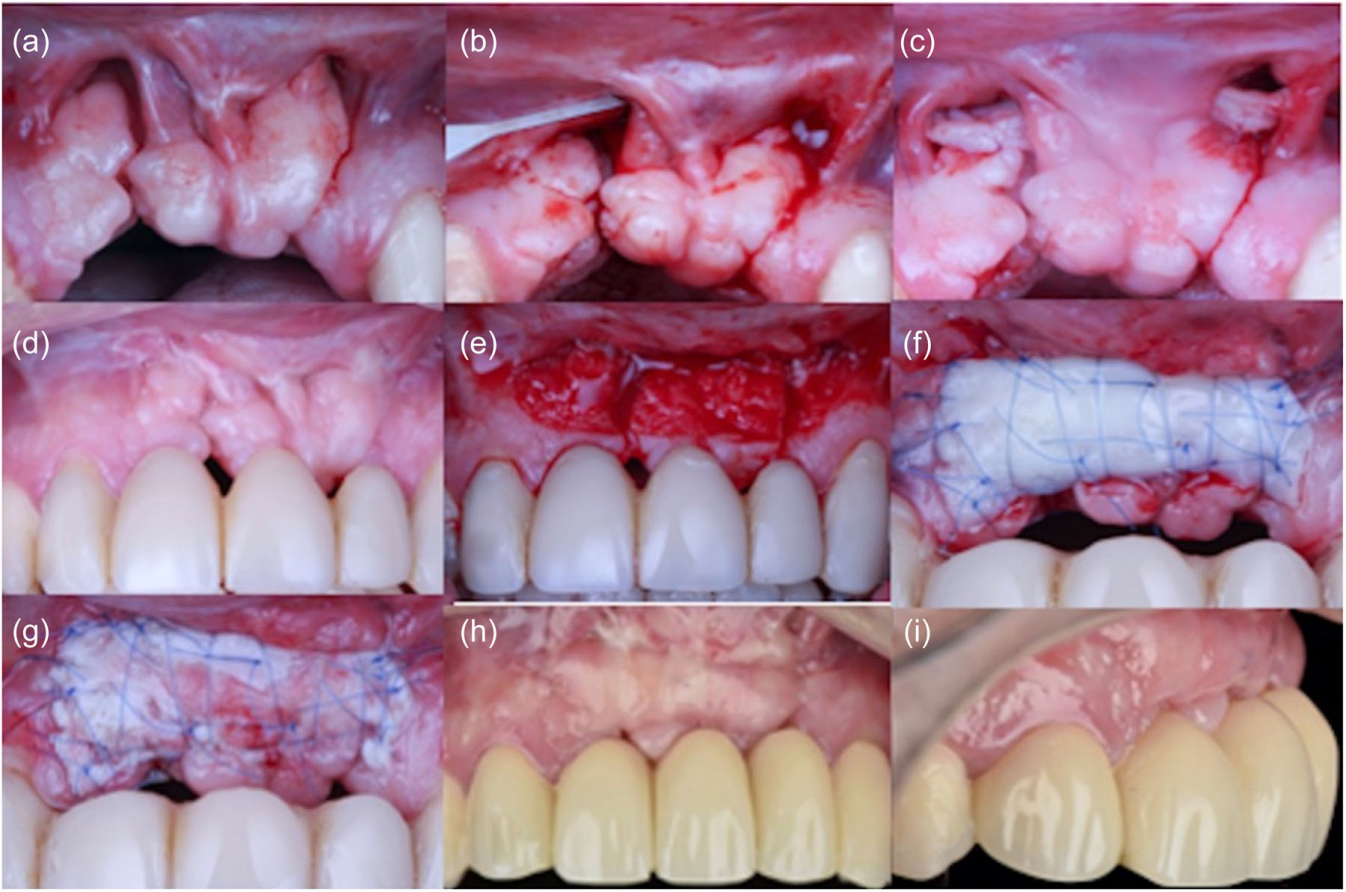
(a) Baseline clinical photo showing bilateral oronasal fistulas; (b) microdissection was performed at split thickness under the fissures and to the base of the nose using an MJK Instruments 003‐004 microblade; (c) connective tissue graft was placed inside the recipient bed and secured with absorbable sutures; (d) 12 weeks after connective tissue graft (CTG); (e) split‐thickness flap was prepared for free gingival graft (FGG) augmentation (f); (g) 2 weeks after FGG healing; (h, i) first‐stage healing.

**Figure 3:**
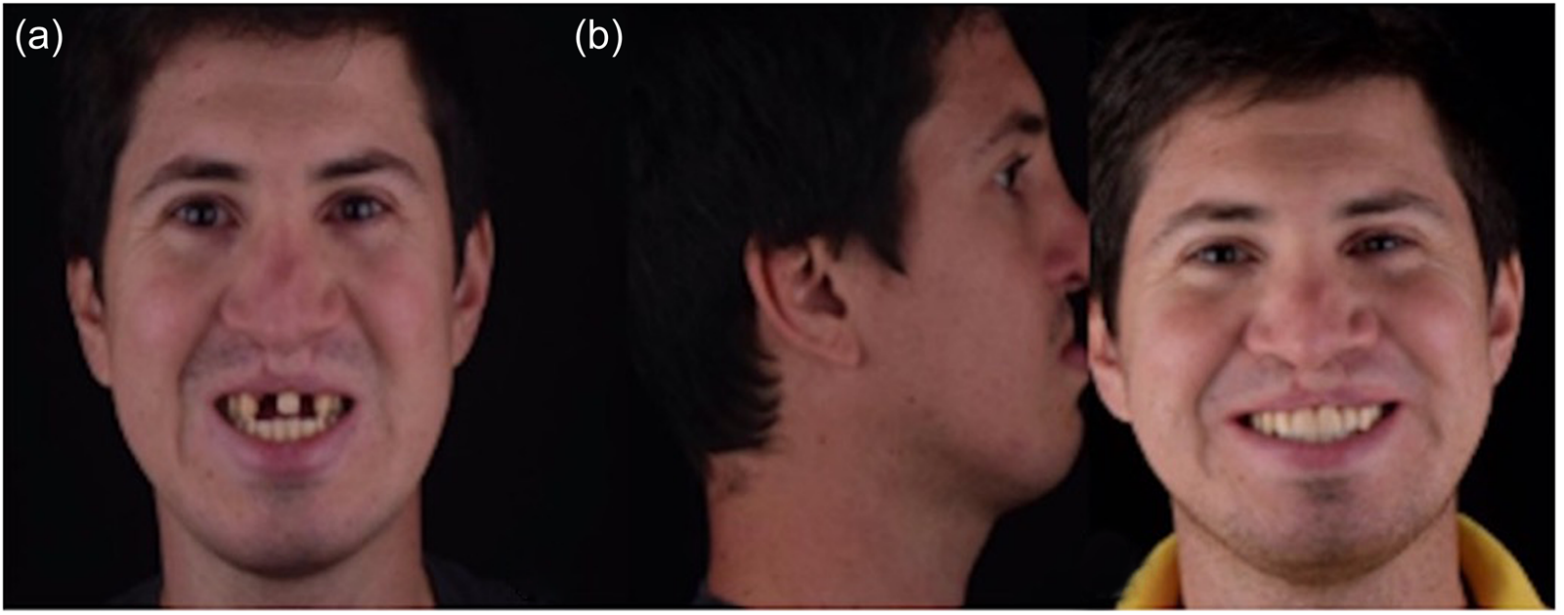
(a) Before and (b) after patient face and smile.

**Figure 4:**
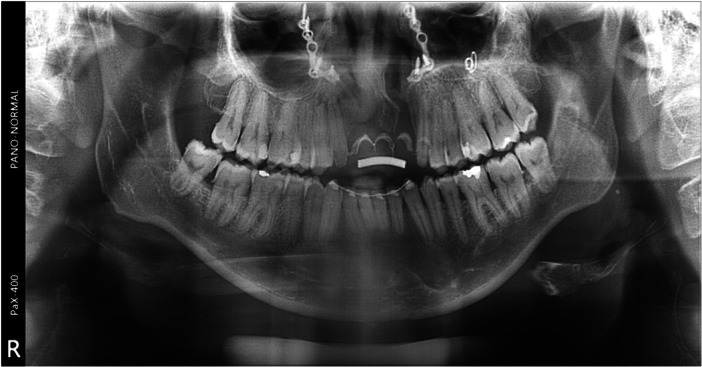
A panoramic radiograph after 36 months of follow‐up, showcasing the previous surgical repair of bilateral cleft lip and palate with oronasal fistula.

In the second stage, following 6 months of uneventful healing after the fistulas had closed, the recipient bed was prepared, encompassing the area down to the bottom of the vestibule and involving a neighboring tooth. Microsurgical blades and a 15c surgical blade were used for this procedure. A free gingival graft, harvested from the palate, was affixed in this prepared area using microsurgical suture mesh and 6‐0 Polypropylene (Figure 2a–i). Finally, the same postoperative measures as in Stage 1 were indicated (Figure 3a,b). At 36 months of follow‐up, a panoramic radiograph demonstrates the previous surgical repair of bilateral cleft lip and palate with oronasal fistula (Figure 4).

## 4 Case 2

### 4.1 Clinical Presentation

A 30‐year‐old patient in good health, who does not smoke, was referred for a consultation at Dr. Chacon's Private Practice in Medellin, Colombia. The patient sought maxillofacial surgery to address the consequences of a prior Giant Cell Granuloma, which had occurred several years earlier. As a result of this condition, the patient had lost her central and lateral incisors and developed an oronasal communication related to a vertical defect. Her complaints were similar to those described in Case No. 1, including regurgitation, discomfort, breathing difficulties, unpleasant odors, and aesthetic concerns that significantly impacted her quality of life. After being informed about the proposed treatment plan, she agreed to proceed with the staged approach.

### 4.2 Surgical Case Management

**Figure 5:**
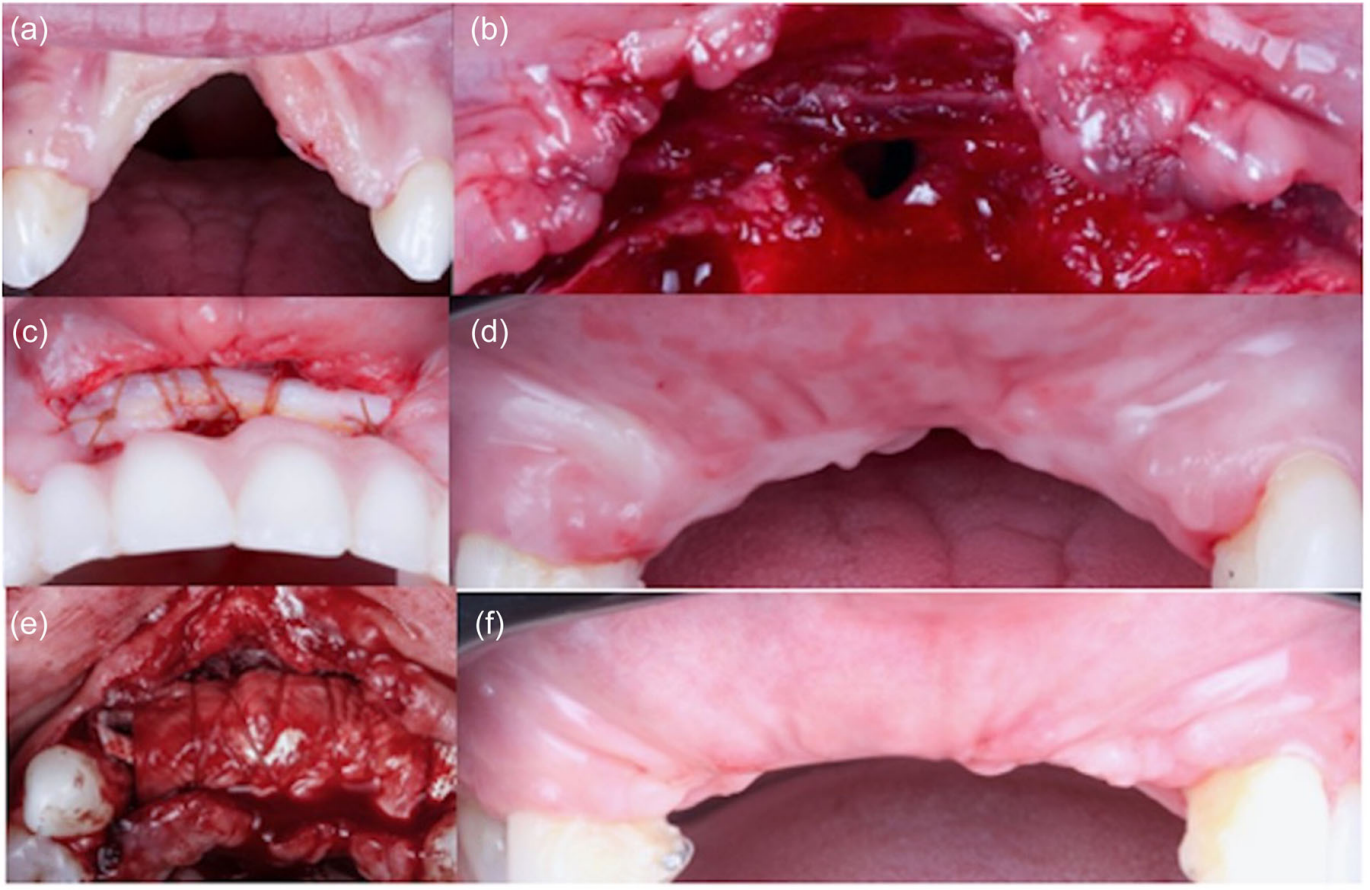
(a, b) Baseline related to a complex anterior vertical defect and oronasal communication; (c) CTG fixed over the fistula to close using Catgut 6‐0; (d) first‐stage healing; (e) vertical ridge augmentation; (f) 6‐month second‐stage healing.

In Stage 1 of the treatment, two vertical releasing incisions were made, starting mesially from one adjacent tooth to the defect. A full‐thickness flap was raised in the area of the defect (oronasal communication). Additionally, partial‐thickness areas were created as tunnels to ensure an adequate blood supply. A connective tissue graft, harvested from the lateral palate, was secured in the area using absorbable sutures (Catgut 6‐0) at the edges, and a mesh was placed over the graft (Figure 5a–d). Following periosteum release and flap advancement, external closure was performed using 6‐0 Polypropylene sutures. After a 6‐month period with no adverse events, vertically guided bone regeneration was carried out. This procedure followed the surgical principles described by Urban et al. (2023), which include a safety flap, subperiosteal preparation, papilla's repositioning, the use of Ti‐reinforced membranes (Cytoplast, Osteogenics, USA), and a combination of xenograft (Bio‐oss, Geistlich Pharma North America Inc., NJ, USA) and autogenous bone (see Figure 5e,f).

**Figure 6:**
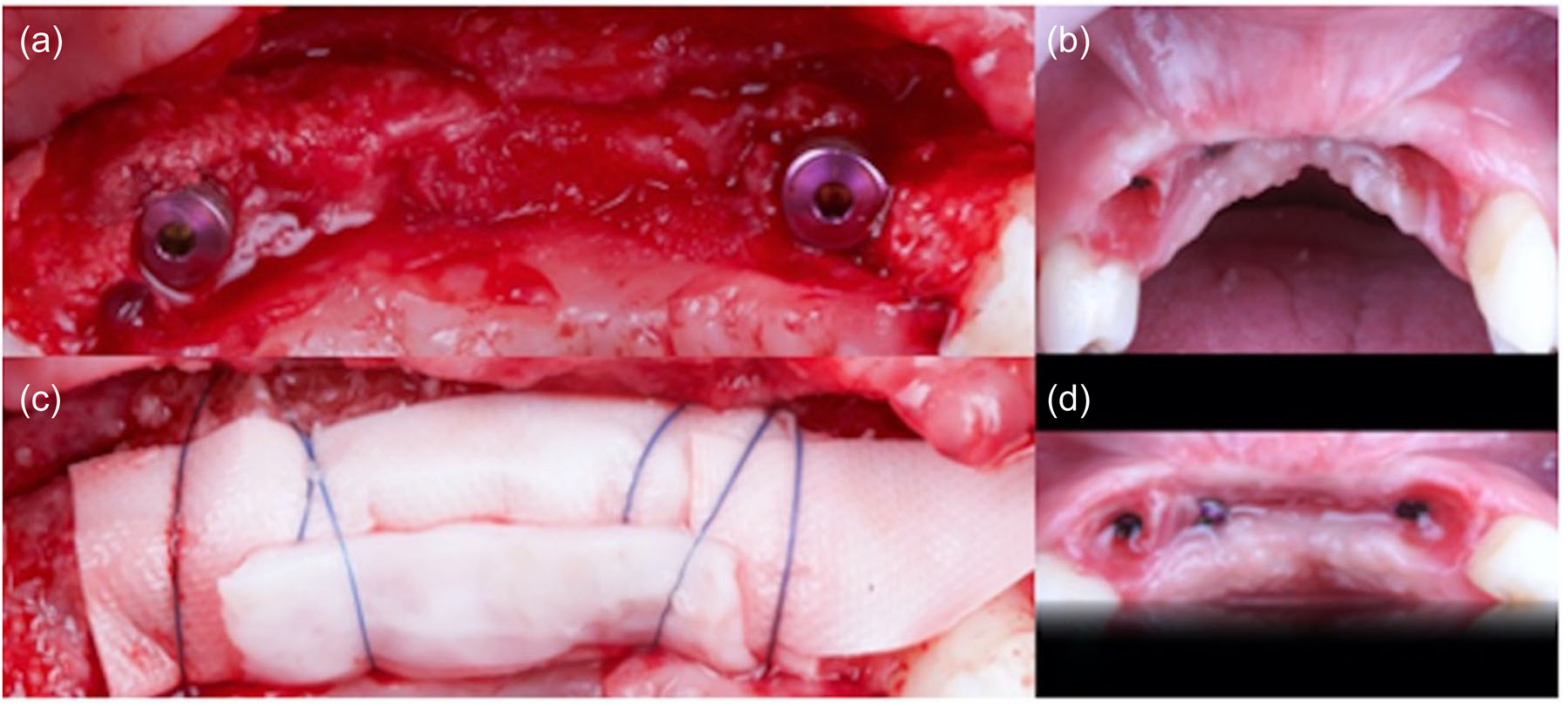
(a, b) Third‐stage surgery (after fistula closure and vertical GBR) with implant insertion; (c) bone augmentation using a double layer of long‐term resorbable membrane and fixed with 6‐0 Polypropylene; (d) final healing results.

**Figure 7:**
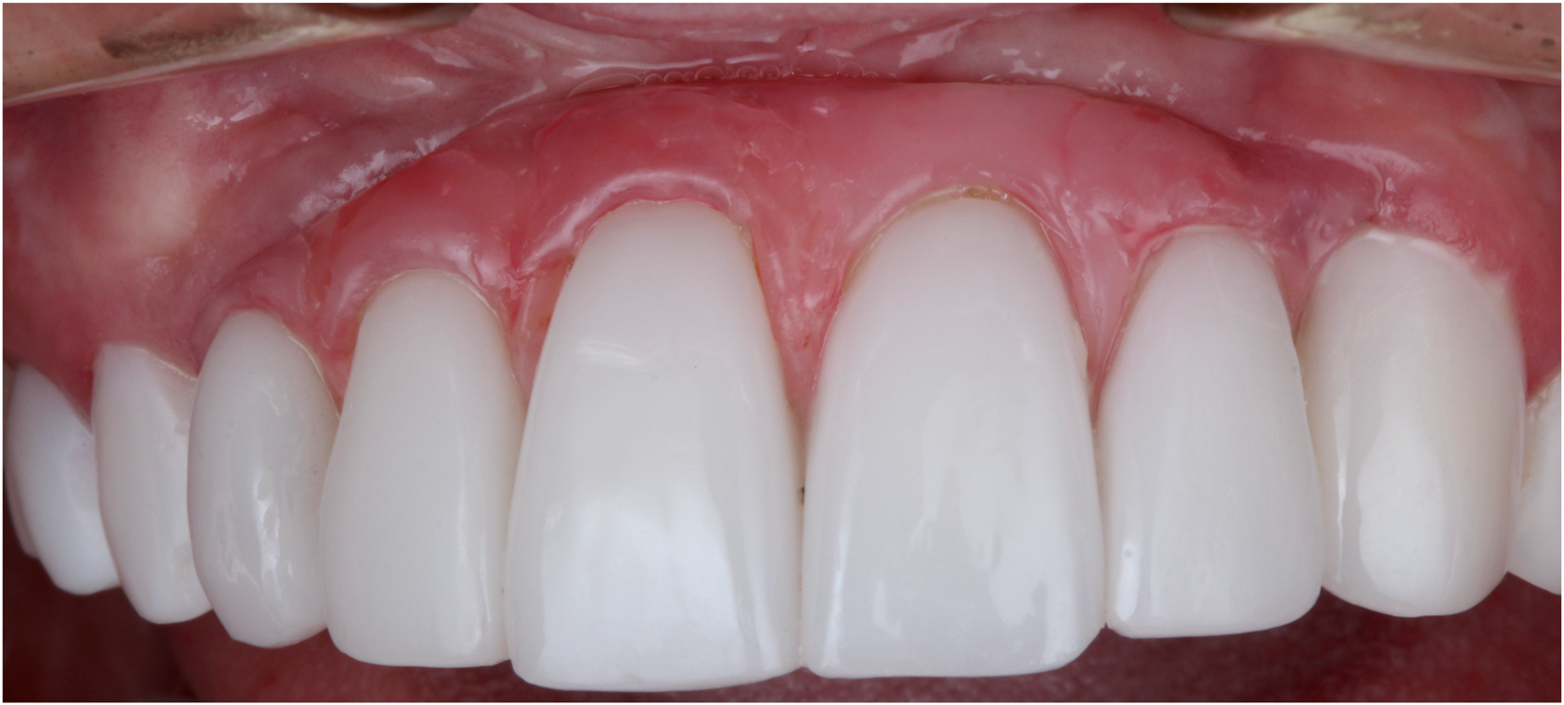
Close‐up view of the final restoration after 36 months of follow‐up.

**Figure 8:**
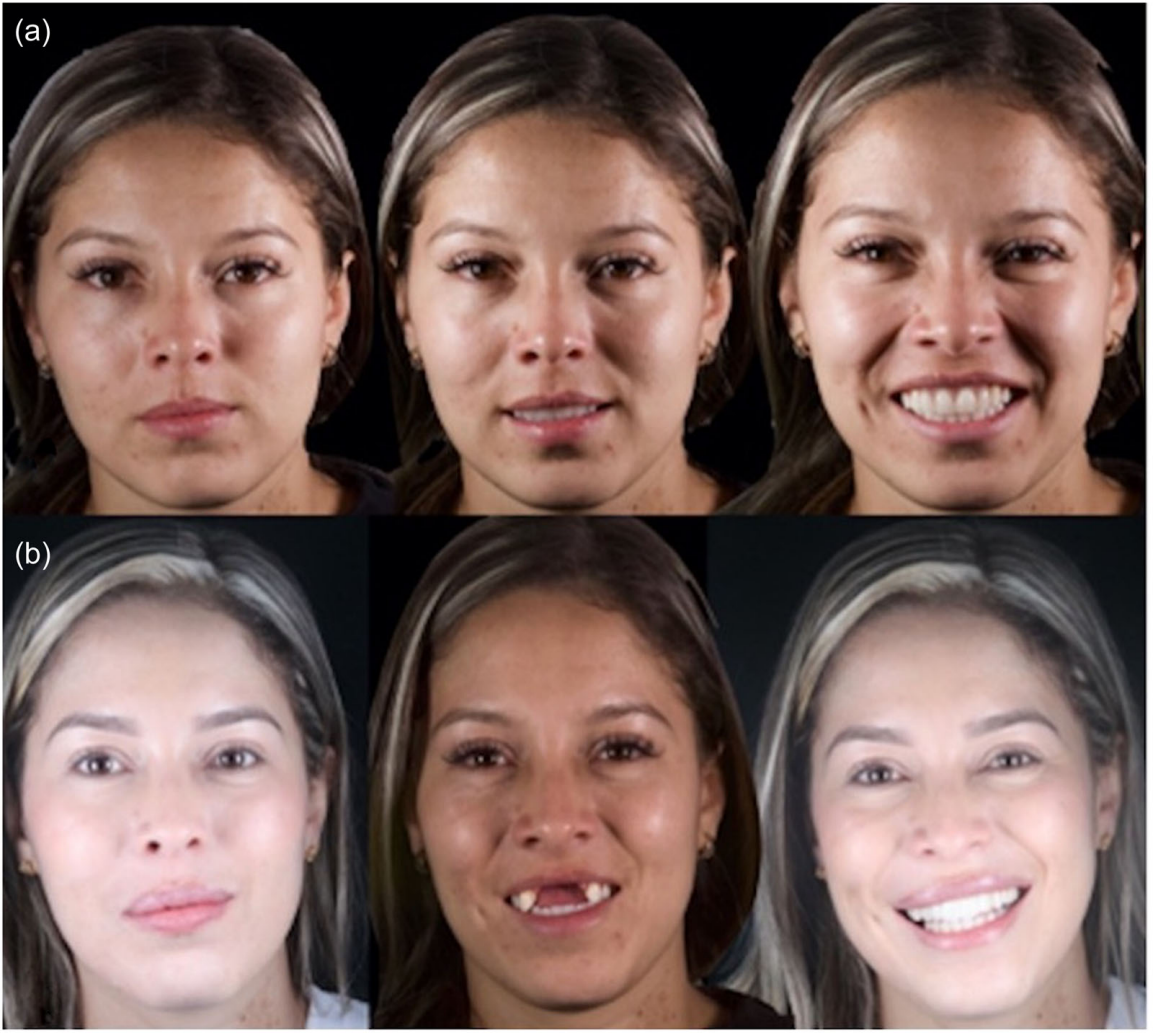
Patient's (a) facial and (b) smile photos from baseline to final results.

**Figure 9:**
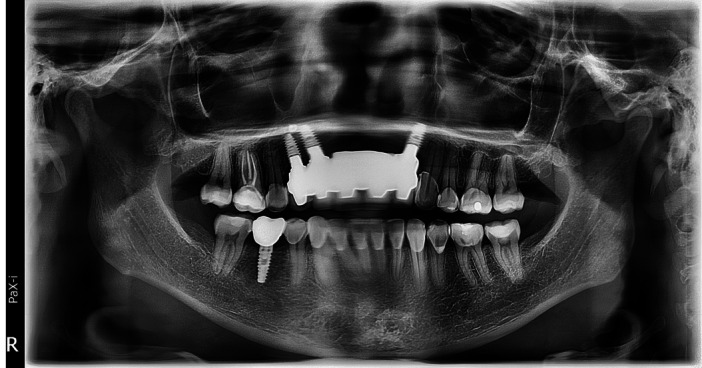
A panoramic radiograph after 36 months of follow‐up, showcasing the surgical repair for the vertical defect and oronasal communication, along with implants with stable bone.

Around 7 months later, the membrane was removed due to exposure and the presence of pus. The area was cleaned with saline solution and chlorhexidine, followed by the insertion of implants and additional bone augmentation using cross‐linked membranes (Ossix Plus, Datum Dental, Dentsply Sirona, USA) and xenograft (Bio‐oss, Geistlich Pharma North America Inc., NJ, USA). After a smooth 6‐month healing period, the final rehabilitation was performed (Figure 6a–d). The patient's progress has been positive with no adverse events, and she expresses complete satisfaction with both clinical and radiographical outcomes, even beyond 3 years following the procedure (Figures 7 and 8a,b) and (Figure 9).

The patient received postsurgical instructions, which included rinsing with saline solution three times a day, taking 500 mg of amoxicillin every 8 h, and 600 mg of ibuprofen every 8 h for 1 week, as in Case No. 1.

## 5 Results

During Stage 1, the patient underwent regular check‐ups at 24 h, 72 h, 5 days, and 7 days after surgery. Subsequently, follow‐up appointments were scheduled once a week for the first 3 months. These follow‐up visits involved monitoring the hygiene of the treated area and rinsing with saline solution. The external sutures were removed 2 weeks after the procedure.

For Stage 2 of the first case and Case No. 2, a similar follow‐up protocol was followed, and the sutures were also removed after 2 weeks. Notably, no adverse events were observed in either stage.

Clinically for both cases, during Stage 1, there were no signs of disruption or perforation in the surgical bed that was created. The closure was complete, and the graft remained intact. From a clinical perspective, it was possible to confirm the closure of both fistulas, leading to noticeable improvements in the patient's breathing, phonation, and the closure of both fistulas.

In Stage 2 of both cases, there was a 100% adaptation of the graft, with its integrity, survival, and vascularity becoming evident within 24–72 h. The area displayed an improved phenotype, thickness, closure, and aesthetics compared to the previous vestibular fissures. Over a follow‐up period exceeding 36 months for both cases, the stability of the treated area remained evident. The patient reported significant enhancements in their quality of life, including improvements in breathing, phonation, and feeding.

## 6 Discussion and Conclusion

Nasopalatine clefts pose a significant challenge in the field of maxillofacial surgery and necessitate early intervention within the first days or months of a patient's life to enhance their chances and overall quality of life (Cohen et al. 1991). However, while managing this congenital condition, complications and aftereffects may arise. One such complication is the development of oronasal fistulas. These fistulas are characterized by the presence of an epithelialized tract that connects the nasal and oral structures. They result in discomfort affecting speech, breathing, food intake, and even the personal and social aspects of a patient's life (Cohen et al. 1991). Consequently, the presence of these sequelae hinders the achievement of the desired surgical outcomes (Li, Yin, and Song 2015; Posnick and Getz 1987). Although nasopalatine clefts can be classified and managed with guidance, the early occurrence of fistulas can often be attributed to critical technical factors. These factors include inadequate mobilization of the flap, excessive tension during closure, improper displacement, issues with blood supply, perforations, suboptimal tissue handling, graft necrosis, inappropriate suture selection, and postoperative infections (Posnick and Getz 1987; Rohrich et al. 1996). Additionally, research suggests that the occurrence of postoperative fistulas is more strongly associated with two‐stage maxillofacial procedures in pediatric patients, as observed in this report (Pollard et al. 2021; Zucchelli et al. 2020).

Similarly, when addressing the correction of remaining lip–palatal cleft fistulas, there is a wide array of techniques available for closure management. These techniques include obturators and various types of grafts, as well as lingual, palatal rotational, displaced, or vestibular flaps, as previously mentioned. Each of these techniques has its own set of advantages and limitations, but they all share a common goal of enhancing the patient's quality of life, particularly in terms of breathing, eating, and speech. A key objective shared by all reconstructive techniques is to establish a permanent separation between the oral and nasal cavities.

Conversely, the treatment and surgical management of anterior oronasal fistulas pose a greater challenge due to various factors. These factors include the thin periodontal tissue, the often‐absent vestibular table (in cases like these), the frenulum, labial musculature, compromised blood supply, and the high aesthetic demands of the patients (Li, Yin, and Song 2015). Over the years, there has been a growing body of scientific evidence supporting the use of periodontal plastic surgery techniques. These techniques involve the microsurgical management of periodontal compartments (epithelium, connective tissue, periosteum, and bone) using magnification and appropriate instruments. Additionally, they entail the use of connective tissue grafts sourced from different areas of the palate. These grafts are utilized to address a range of mucogingival and phenotypic defects associated with teeth and implants. Applications include recession coverage, bone regeneration, and papilla reconstruction. Furthermore, these techniques can be adapted for the management and resolution of complex defects following maxillofacial surgeries, as demonstrated in this case report (Barootchi et al. 2020; Chacón et al. 2023; Chacón R and Retana 2022; Tavelli et al. 2018, 2019; Zadeh 2011; Zuhr et al. 2018).

For the authors' better understanding, this approach offers several advantages over other techniques described in recent literature, such as tongue flaps, multilayered closure, intra and extraoral nasal flaps, and the endoscopic approach. These advantages stem from four main features: First, these techniques are rooted in microsurgical principles involving partial‐to‐total thickness compartments, tunnels, and coronal advance flaps, which are less complex and invasive than a tongue flap. Second, these techniques can be performed in a dental office environment without the need for complex devices beyond magnification, microsurgical instruments, and sutures. Third, the number of interventions required could range from 2 to 3, with maximum surgeries including inner closure of the communication using connective tissue grafts, guided bone regeneration, and a free gingival graft for the final improvement of the phenotype. Finally, this approach directly addresses the final aesthetic desires of patients, aligning with their aesthetic goals and improving overall satisfaction (Kim and Seo 2023; Meneses Argalle, Espinosa Orozco, and Prada Madrid 2023; Obermeyer et al. 2022; Zielinska‐Kazmierska et al. 2021).

Clefts of the lip and palate pose a significant and intricate challenge for maxillofacial surgery and necessitate a comprehensive management plan. The presence of oronasal fistulas resulting from corrective Maxillofacial surgery demands identification and treatment which encompass various factors, including considerations related to breathing, feeding, and aesthetics. Despite the constraints of this article, it is worth noting that periodontal plastic surgery procedures, coupled with the use of connective tissue grafts, offer a viable approach to addressing oronasal fistulas. These techniques hold promise for enhancing the patient's quality of life and improving the aesthetics of the affected area.

## Author Contributions

Study conception and design: G.C., H.R., and A.A. Data collection: G.C. and H.R. Drafting of the manuscript: G.C., A.A., O.M., A.A‐L., and H‐L.W. All authors gave their final approval and agreed to be accountable for all aspects of the work.

## Consent

A written consent was provided to the author regarding this case series. However, no patient identifiers were used.

## Conflicts of Interest

The authors declare no conflicts of interest.

## Supporting information

Supporting information.

Supporting information.

## Data Availability

Original raw data collected and the data that support the findings of this study are available from the corresponding author upon reasonable request.

## References

[cre2914-bib-0001] Abdel‐Aziz, M. 2010. “V–Y Two‐Layer Repair for Oronasal Fistula of Hard Palate.” International Journal of Pediatric Otorhinolaryngology 74: 1054–1057. 10.1016/j.ijporl.2010.06.003. 20591506

[cre2914-bib-0002] Abuabara, A. , A. L. V. Cortez , L. A. Passeri , M. de Moraes , and R. W. F. Moreira . 2006. “Evaluation of Different Treatments for Oroantral/Oronasal Communications: Experience of 112 Cases.” International Journal of Oral and Maxillofacial Surgery 35: 155–158. 10.1016/j.ijom.2005.04.024. 15955666

[cre2914-bib-0003] Aznar, M. L. , B. Schönmeyr , G. Echaniz , L. Nebeker , L. Wendby , and A. Campbell . 2015. “Role of Postoperative Antimicrobials in Cleft Palate Surgery: Prospective, Double‐Blind, Randomized, Placebo‐Controlled Clinical Study in India.” Plastic and Reconstructive Surgery 136: 59e–66e. 10.1097/PRS.0000000000001324. 26111333

[cre2914-bib-0004] Barootchi, S. , L. Tavelli , G. Zucchelli , W. V. Giannobile , and H. L. Wang . 2020. “Gingival Phenotype Modification Therapies on Natural Teeth: A Network Meta‐Analysis.” Journal of Periodontology 91: 1386–1399. 10.1002/JPER.19-0715. 32392401

[cre2914-bib-0005] Bonanthaya, K. , P. Shetty , A. Sharma , J. Ahlawat , D. Passi , and M. Singh . 2016. “Treatment Modalities for Surgical Management of Anterior Palatal Fistula: Comparison of Various Techniques, Their Outcomes, and the Factors Governing Treatment Plan: A Retrospective Study.” National Journal of Maxillofacial Surgery 7: 148–152. 10.4103/0975-5950.201357. 28356685 PMC5357918

[cre2914-bib-0006] Chacón, G. , M. H. A. Saleh , C. Fleming , N. Leon , and H. L. Wang . 2023. “Papilla Reconstruction for an Iatrogenic RT3 Gingival Defect Using a Tuberosity Soft Tissue Graft: A Case Report.” Clinical Advances in Periodontics 13: 163–167. 10.1002/cap.10233. 36636761

[cre2914-bib-0007] Chacón R, G. J. , and L. Retana . 2022. “The Connective Tissue Graft as a Membrane to Improve Esthetics According the Defect.” Journal of Stomatology, Oral and Maxillofacial Surgery 123: 514–520. 10.1016/j.jormas.2022.04.012. 35569726

[cre2914-bib-0008] Cohen, S. R. , J. Kalinowski , D. LaRossa , and P. Randall . 1991. “Cleft Palate Fistulas: A Multivariate Statistical Analysis of Prevalence, Etiology, and Surgical Management.” Plastic and Reconstructive Surgery 87: 1041–1047. 2034725

[cre2914-bib-0010] Honnebier, M. B. O. M. , D. S. Johnson , A. A. Parsa , A. Dorian , and F. D. Parsa . 2000. “Closure of Palatal Fistula With a Local Mucoperiosteal Flap Lined With Buccal Mucosal Graft.” The Cleft Palate‐Craniofacial Journal 37: 127–129. 10.1597/1545-1569_2000_037_0127_copfwa_2.3.co_2. 10749052

[cre2914-bib-0011] Kim, B. Y. , and B. F. Seo . 2023. “Endoscope‐Assisted Multilayered Repair in Oronasal Fistula.” Ear, Nose & Throat Journal 102, no. 4 (April): 268–271. 10.1177/0145561321997607 33634719

[cre2914-bib-0012] Li, H. , N. Yin , and T. Song . 2015. “Oronasal Fistula Repair Using the Alveolar Ridge Approach.” International Journal of Pediatric Otorhinolaryngology 79: 161–164. 10.1016/j.ijporl.2014.11.033. 25542863

[cre2914-bib-0013] MacLeod, A. M. , W. A. Morrison , J. J. McCann , S. Thistlethwaite , C. A. Vanderkolk , and A. D. Ryan . 1987. “The Free Radial Forearm Flap With and Without Bone for Closure of Large Palatal Fistulae.” British Journal of Plastic Surgery 40: 391–395. 10.1016/0007-1226(87)90043-9. 3620783

[cre2914-bib-0014] Meneses Argalle, J. D. , A. M. Espinosa Orozco , and J. R. Prada Madrid . 2023. “Tongue Flap for Closure of Complex Oronasal Fistula.” Journal of Craniofacial Surgery 34, no. 6 (September) 1872–1875. 37344931 10.1097/SCS.0000000000009468

[cre2914-bib-0015] Obermeyer, I. P. , N. Sterritt , Y. M. Haidar , T. Tjoa , and E. C. Kuan . 2022. “Multilayered Closure of Oronasal and Oroantral Fistula Using Intranasal and Intraoral Flaps.” The Laryngoscope 132, no. 11 (November): 2259–2261. 35348210 10.1002/lary.30111

[cre2914-bib-0016] Pollard, S. H. , J. R. Skirko , D. Dance , et al. 2021. “Oronasal Fistula Risk After Palate Repair.” The Cleft Palate‐Craniofacial Journal 58, 35–41. 10.1177/1055665620931707. 32573252

[cre2914-bib-0017] Posnick, J. C. , and S. B. Getz Jr. 1987. “Surgical Closure of End‐Stage Palatal Fistulas Using Anteriorly Based Dorsal Tongue Flaps.” Journal of Oral and Maxillofacial Surgery 45: 907–912. 10.1016/0278-2391(87)90438-1. 3478437

[cre2914-bib-0018] Robertson, A. G. N. , D. J. McKeown , G. Bello‐Rojas , et al. 2008. “Use of Buccal Myomucosal Flap in Secondary Cleft Palate Repair.” Plastic and Reconstructive Surgery 122, 910–917. 10.1097/PRS.0b013e318182368e. 18766058

[cre2914-bib-0019] Rohrich, R. J. , A. R. Rowsell , D. F. Johns , et al. 1996. “Timing of Hard Palatal Closure: A Critical Long‐Term Analysis.” Plastic and Reconstructive Surgery 98, 236–246. 10.1097/00006534-199608000-00005. 8764711

[cre2914-bib-0020] Simpson, A. , O. A. Samargandi , A. Wong , M. E. Graham , and M. Bezuhly . 2019. “Repair of Primary Cleft Palate and Oronasal Fistula With Acellular Dermal Matrix: A Systematic Review and Surgeon Survey.” The Cleft Palate‐Craniofacial Journal 56: 187–195. 10.1177/1055665618774028. 29727220

[cre2914-bib-0021] Smith, D. M. , L. Vecchione , S. Jiang , et al. 2007. “The Pittsburgh Fistula Classification System: A Standardized Scheme for the Description of Palatal Fistulas.” The Cleft Palate‐Craniofacial Journal 44, no. 6: 590–594. 10.1597/06-204.1. 18177198

[cre2914-bib-0022] Steele, M. H. , and M. B. Seagle . 2006. “Palatal Fistula Repair Using Acellular Dermal Matrix: The University of Florida Experience.” Annals of Plastic Surgery 56: 50–53. 10.1097/01.sap.0000185469.80256.9e. 16374096

[cre2914-bib-0023] Tavelli, L. , S. Barootchi , F. Cairo , G. Rasperini , K. Shedden , and H. L. Wang . 2019. “The Effect of Time on Root Coverage Outcomes: A Network Meta‐Analysis.” Journal of Dental Research 98: 1195–1203. 10.1177/0022034519867071. 31381868

[cre2914-bib-0024] Tavelli, L. , S. Barootchi , T. V. N. Nguyen , M. Tattan , A. Ravidà , and H. L. Wang . 2018. “Efficacy of Tunnel Technique in the Treatment of Localized and Multiple Gingival Recessions: A Systematic Review and Meta‐Analysis.” Journal of Periodontology 89: 1075–1090. 10.1002/JPER.18-0066. 29761502

[cre2914-bib-0025] Tunçbilek, G. , E. Konaş , A. Kayikçioğlu , and E. M. Mavili . 2012. “Three‐Layer Oronasal Fistula Repair With Sandwiched Mastoid Fascia Graft.” Journal of Craniofacial Surgery 23: 780–783. 10.1097/SCS.0b013e31824dbd68. 22565897

[cre2914-bib-0026] Urban, I. , E. Montero , I. Sanz‐Sánchez , et al. 2023. “Minimal Invasiveness in Vertical Ridge Augmentation.” Periodontology 2000 91: 126–144. 10.1111/prd.12479. 36700299

[cre2914-bib-0027] Wilhelmi, B. J. , E. A. Appelt , L. Hill , and S. J. Blackwell . 2001. “Palatal Fistulas: Rare With the Two‐Flap Palatoplasty Repair.” Plastic and Reconstructive Surgery 107: 315–318. 10.1097/00006534-200102000-00002. 11214043

[cre2914-bib-0028] Zadeh, H. H. 2011. “Minimally Invasive Treatment of Maxillary Anterior Gingival Recession Defects by Vestibular Incision Subperiosteal Tunnel Access and Platelet‐Derived Growth Factor BB.” The International Journal of Periodontics & Restorative Dentistry 31: 653–660. 22140667

[cre2914-bib-0029] Zhang, B. , J. Li , D. Sarma , F. Zhang , and J. Chen . 2014. “The Use of Heterogeneous Acellular Dermal Matrix in the Closure of Hard Palatal Fistula.” International Journal of Pediatric Otorhinolaryngology 78: 75–78. 10.1016/j.ijporl.2013.10.053. 24290949

[cre2914-bib-0030] Zielinska‐Kazmierska, B. , J. Grodecka , W. Lucas Grzelczyk , and M. Jozefowicz‐Korczynska . 2021. “Long‐Term Results of Three‐Layered Closure of Oronasal Fistula: A Case Report.” Plastic and Reconstructive Surgery—Global Open 9 (December): 3964. 10.1097/GOX.0000000000003964 PMC8683243 34934600

[cre2914-bib-0031] Zucchelli, G. , L. Tavelli , M. K. McGuire , et al. 2020. “Autogenous Soft Tissue Grafting for Periodontal and Peri‐Implant Plastic Surgical Reconstruction.” Journal of Periodontology 91, 9–16. 10.1002/JPER.19-0350. 31461778

[cre2914-bib-0032] Zuhr, O. , S. F. Rebele , S. L. Cheung , and M. B. Hürzeler . 2018. “Surgery Without Papilla Incision: Tunneling Flap Procedures in Plastic Periodontal and Implant Surgery.” Periodontology 2000 77: 123–149. 10.1111/prd.12214. 29493018

