# Supplementary figures and images for: Oronasal Fistula Closure and Defect Reconstruction: Two Case Reports Using Periodontal Plastic Surgery Principles
Source: Clin Exp Dent Res. 2024 Jul 7;10(4):e914. doi: 10.1002/cre2.914 (PMC11228353; doi:10.1002/cre2.914)

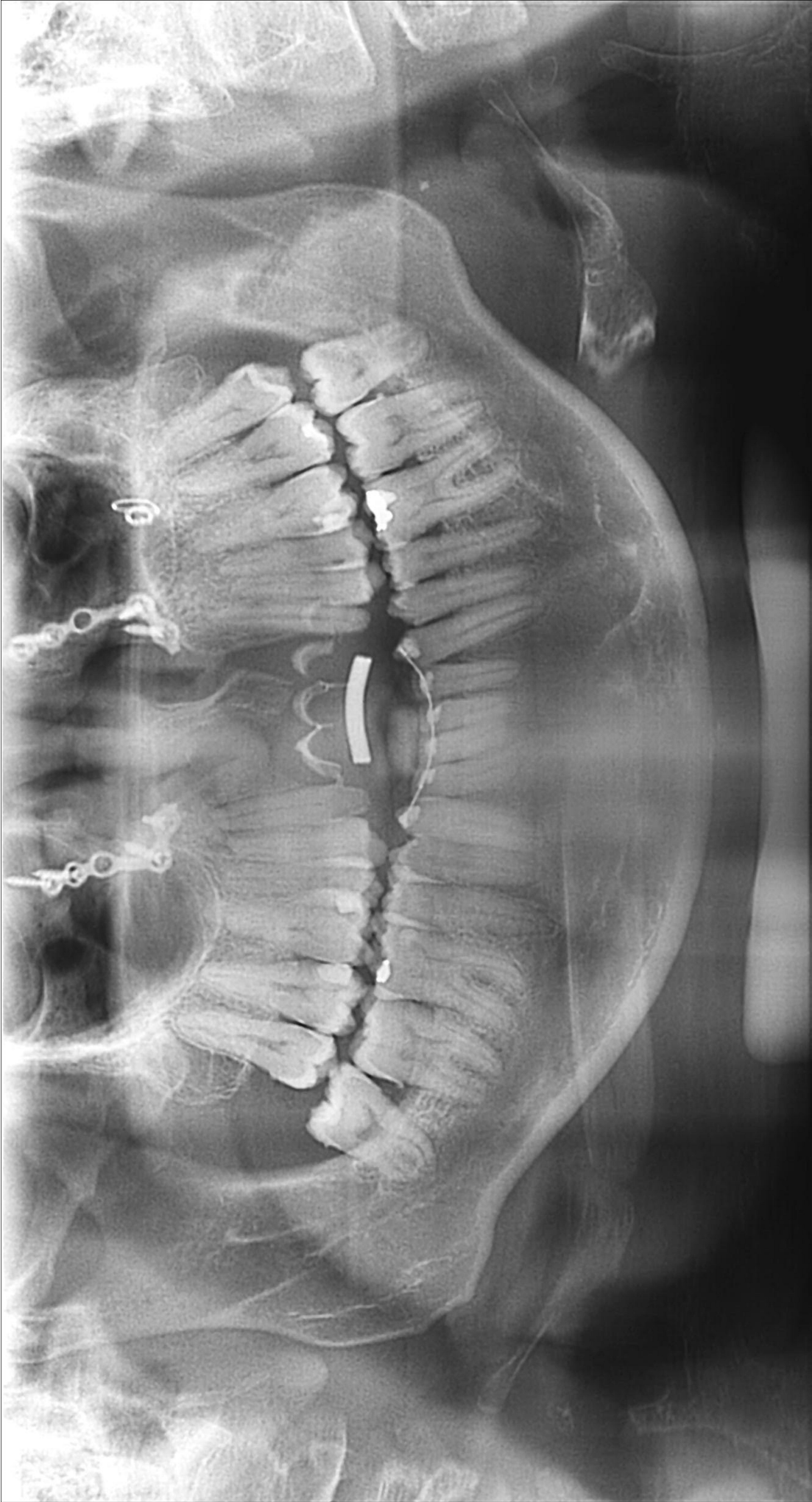

Supplement: Supplementary file 1 — Supporting information. [file CRE2-10-e914-s001.pdf]

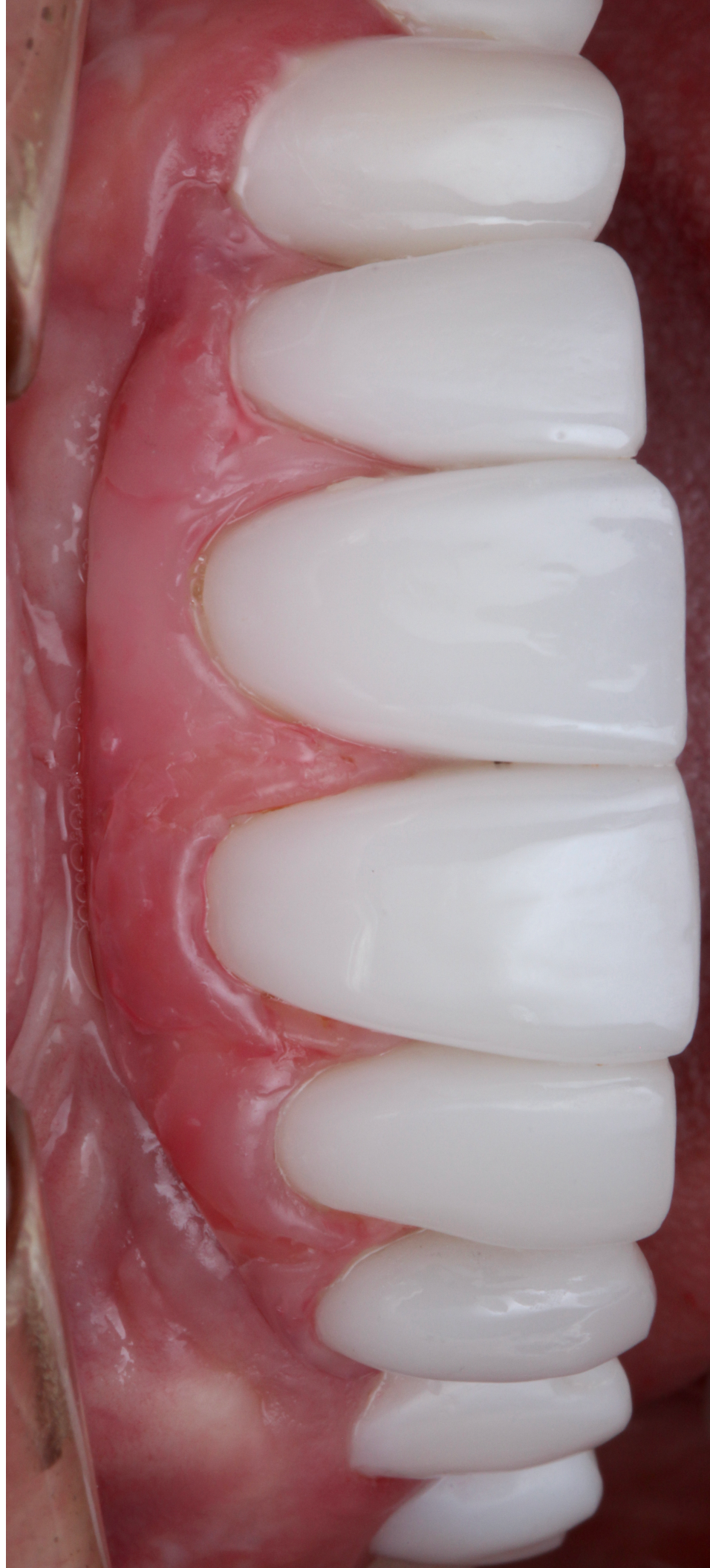

Supplement: Supplementary file 2 — Supporting information. [file CRE2-10-e914-s002.pdf]
